# Fibrin(ogen) as a Therapeutic Target: Opportunities and Challenges

**DOI:** 10.3390/ijms22136916

**Published:** 2021-06-28

**Authors:** Thembaninkosi G. Gaule, Ramzi A. Ajjan

**Affiliations:** Division of Cardiovascular & Diabetes Research, Leeds Institute of Cardiovascular and Metabolic Medicine (LICAMM), University of Leeds, Leeds LS2 9JT, UK; t.g.gaule@leeds.ac.uk

**Keywords:** fibrinogen, fibrin, fibrinolysis, thrombosis, hyperfibrinolysis, therapeutics

## Abstract

Fibrinogen is one of the key molecular players in haemostasis. Thrombin-mediated release of fibrinopeptides from fibrinogen converts this soluble protein into a network of fibrin fibres that form a building block for blood clots. Thrombin-activated factor XIII further crosslinks the fibrin fibres and incorporates antifibrinolytic proteins into the network, thus stabilising the clot. The conversion of fibrinogen to fibrin also exposes binding sites for fibrinolytic proteins to limit clot formation and avoid unwanted extension of the fibrin fibres. Altered clot structure and/or incorporation of antifibrinolytic proteins into fibrin networks disturbs the delicate equilibrium between clot formation and lysis, resulting in either unstable clots (predisposing to bleeding events) or persistent clots that are resistant to lysis (increasing risk of thrombosis). In this review, we discuss the factors responsible for alterations in fibrin(ogen) that can modulate clot stability, in turn predisposing to abnormal haemostasis. We also explore the mechanistic pathways that may allow the use of fibrinogen as a potential therapeutic target to treat vascular thrombosis or bleeding disorders. Better understanding of fibrinogen function will help to devise future effective and safe therapies to modulate thrombosis and bleeding risk, while maintaining the fine balance between clot formation and lysis.

## 1. Introduction

Fibrinogen is one of the most abundant plasma proteins, circulating at 2–3 mg/mL concentrations, but levels can more than double in pathological states [1,2]. Soluble fibrinogen is converted into an insoluble fibrin network, which forms the backbone of the blood clot and has a critical role in haemostasis by limiting blood loss following vascular injury [3]. However, in diseased blood vessels, the rupture of an atheromatous plaque can trigger pathological clot formation, which, in severe cases, blocks the vessel, causing end organ damage including myocardial infarction and stroke.

Quantitative and qualitative changes in fibrinogen can result in fibrin networks that are difficult to breakdown [4], thus increasing the risk of thrombosis and vascular occlusion. Other alterations in fibrinogen can result in ineffective or unstable fibrin networks, thus increasing the risk of bleeding [5]. Therefore, the manipulation of the fibrinogen molecule has the potential to alter thrombosis or bleeding risk by inhibiting clot formation/facilitating lysis or by making clots that are resistant to breakdown.

While the fibrin network is targeted to treat vascular occlusion, there is no treatment directed at the fibrinogen molecule itself. The same applies for conditions associated with blood loss; fibrin sealants, composed of a mixture of coagulation proteins, have been used to reduce bleeding following vascular injury [6], but again, the fibrinogen molecule is not used as a target for bleeding disorders.

In the current review, we describe the process of clot formation and lysis, discuss the factors responsible for stabilising the fibrin network and explore the potential role of the fibrinogen molecule as a therapeutic target.

### Structure of Fibrinogen

**Figure 1:**
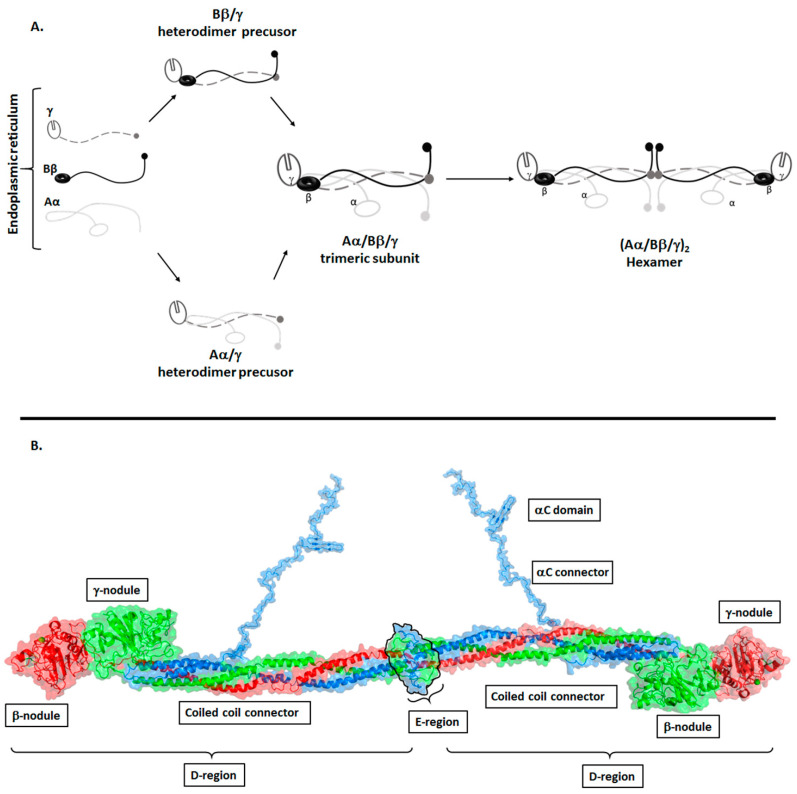
Assembly and structure of fibrinogen. (**Panel A**) Production of fibrinogen in hepatocytes. Once synthesised, fibrinogen chains Aα, Bβ and γ assemble in a stepwise manner. The Aα/γ and Bβ/γ heterodimers are formed first, followed by the (Aα/Bβ/γ) trimeric subunit. Once the trimeric subunits are formed, they dimerise in an antiparallel fashion to form the (Aα/Bβ/γ)_2_ hexamer. (**Panel B**) shows a model of the fibrinogen structure based on the crystal structure of fibrinogen (PDB:3ghg) and NMR structure of the αC domain (PDB:2BAF). The assembly of the (Aα/Bβ/γ)_2_ hexamer gives rise to five regions, the E region, two D regions and two αC regions. The E region is the central nodule that comprises of the N-termini of all the chains (Aα shown in blue, Bβ shown in green and γ shown in red). The D region comprises of a triple coiled coil connector and the β- and γ- nodules. The αC domain composed of the Aα chain and comprises of the αConnector and αC domain.

Fibrinogen circulates in blood as a 340 KDa soluble homodimeric glycoprotein. Each subunit comprises of three polypeptide chains: Aα, Bβ, and γ encoded by three genes, *FGA*, *FGB* and *FGG*, respectively, which are located in a 3-gene cluster on human chromosome 4 [7]. The Aα and Bβ chains are constitutively expressed with their expression regulated by housekeeping mechanisms so as to maintain the levels of circulating fibrinogen in the blood [8,9]. The Bβ chain is transcribed from eight exons and encodes for one form of the Bβ chain. The Aα is transcribed from five exons, however, alternative splicing from a sixth exon encodes for AαE chain, which accounts for 1–3% of circulating fibrinogen [7]. Similar to the Aα chain, γ chains exist in two forms: γ and γ′. The major γ chain is transcribed from ten exons, while intron 9 is retained in γ′ making its C-terminus 20 amino acids longer than the γ chain. Fibrinogen molecules containing γ’ exist as heterodimers γ/γ’ or homodimers γ′/γ′ accounting for 8–15% and <1%, respectively, of the total circulating fibrinogen in healthy individuals [7,10]. The Aα, Bβ and γ chains are expressed, assembled and secreted by hepatocytes as a hexamer (Aα, Bβ, γ)_2_ [11]. Fibrinogen chains are cotranslated into the lumen of the endoplasmic reticulum (ER) where folding and assembly is driven by the primary sequence with assistance of chaperones such as Bip and glycosylation enzymes [2]. Glycosylation begins in the ER and is finalized in the Golgi apparatus, where N-glycosylation of Bβ and γ is completed [2]. Before forming the full (Aα, Bβ, γ)_2_ molecule, each subunit assembles via heterodimer precursors, Aα/γ, Bβ/γ, to form half molecules where the Aα, Bβ and γ chains form triple helical coiled-coils, which are held together by disulphide bonds [2,12] (Figure 1, panel A). Approximately 77% of synthesized fibrinogen is folded and secreted into the extracellular domain [2]. Misfolded or misassembled and surplus protein are retained in the ER and eventually undergo degradation by quality control mechanisms (lysosome and proteasome) [2]. Structural studies have shown that fibrinogen (Aα, Bβ, γ)_2_ assembles such that the Aα, Bβ, γ subunits are antiparallel to each other with the N termini of the subunits interacting with each other via disulphide bonds that hold the two trimeric subunits together to form the hexamer [13,14,15,16,17] (Figure 1). As a result of its structural arrangement (Aα, Bβ, γ)_2_, the module consists of five regions; one central E region, two D regions that flank the region E and two outer αC regions (Figure 1, panel B). Region E is the unique center that contains the N-termini of the six polypeptide chains. The D region comprises of a triple helical coiled coil referred to as the coiled-coil connector and the β- and γ-nodules (Figure 1, panel B). The coiled-coil connectors connect region E to the β- and γ-nodules of region D. The αC region consists solely of the C-terminus of the Aα chain and comprises of an αC connector and αC region. Part of the αC connector folds back into the coiled coil connector through an alpha helix.

## 2. The Biological Role of Fibrinogen (Conversion of Fibrinogen to Fibrin)

Fibrinogen is a multifaceted protein with roles in tissue injury, inflammation, angiogenesis, cell migration and cell adhesion [18,19,20,21]. This review will focus on the role of fibrin(ogen) in clot formation and lysis.

The acute phase response initiated by tissue injury can be divided into two distinct phases that serve to restore haemostasis and repair the injury. The first phase involves the formation of a clot to which fibrinogen is a basic building block. The second phase involves a sequence of events that results in the clearing of the clot, termed fibrinolysis. Fibrin goes from being a building block in phase one to becoming a substrate in phase two. The two phases are coordinated in a precise temporal and spatial manner to reinstate haemostasis, control inflammation and promote tissue repair [19]. 

### 2.1. Clot Formation

**Figure 2:**
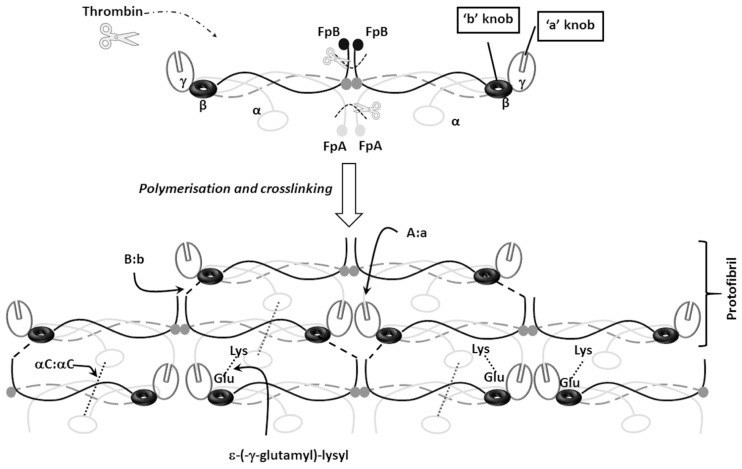
Fibrin polymerisation. Thrombin mediated cleavage of fibrinopeptides FpA and FpB initiates fibrinogen conversion into fibrin. Cleavage of FpA and FpB from the N-termini of Aα and Bβ respectively exposes knob ‘A’ and ‘B’, which in turn interact with complementary pockets ‘a’ and ‘b’ located on the γ- and β- nodules on a neighbouring fibrin monomer. FpB cleavage occurs at slower rate compared to FpA, and this is followed by the release of the αC domains allowing αC:αC interactions and lateral aggregation. Activation of FXIII by thrombin facilitates FXIII-mediated interactions between glutamate (Glu) residues on the γ chain and lysine (Lys) residues on the Aα chain through ε-(γ-glutamyl)-lysyl crosslinks.

In the event of vessel damage because of trauma (external injury), or an atheroma rupture of a diseased vessel (internal injury), blood coagulation is activated through a complex interaction that involves platelets and coagulation factors, culminating in the formation of thrombin. Thrombin initially cleaves off fibrinopeptides A (FpA) followed by cleaving fibrinopeptide B (FpB) from the N-termini of the Aα and Bβ chains, respectively, from fibrinogen, giving rise to fibrin monomers (Figure 2). The thrombin cleavage of FpA exposes a motif (G-P-R), known as knob ‘A’, which interacts with complementary to a pocket ‘a’ located on the γ-nodule of another fibrin monomer [22] (Figure 2). The fibrin monomers spontaneously polymerise into a network of fibres with blood cells embedded in this structure. Polymerisation occurs in two stages, initially the fibrin monomers are organised in a half-staggered and/or double-stranded manner, followed by the protofibrils assembling into fibres through lateral aggregation. FpB is cleaved at a slower rate than FpA, exposing the GHRP motif known as Knob ‘B’ [23]. Knob ‘B’ interacts with a complementary pocket hole ‘b’ in the D region of the β chain on a neighbouring fibrin monomer (Figure 2). Characterisation of the kinetic pathway of the release of FpA and FpB indicated that most of FpB is released after polymerisation has started. Data from biochemical studies have demonstrated that the polymerisation of fibrin can occur in the absence of the ‘B:b’, however, this results in thinner fibrin fibres due to compromised lateral aggregation [23]. Cleavage of FpA and FpB results in conformational change that trigger the release of the αC regions [24,25], which further promotes lateral aggregation through intermolecular αC:αC interactions [24,25,26,27,28] (Figure 2). 

During and after the process of polymerisation, fibrin is covalently crosslinked by thrombin-activated factor XIIIa (FXIIIa) [29], which catalyses the formation of ε- (-γ-glutamyl)-lysyl crosslinks between lysine and glutamate residues in the γ- chain, increasing fibre density and stiffness [30,31,32] (Figure 2). The α- chains crosslinks occur at a much slower rate, traversing between fibrin strands rendering the clot less susceptible to lysis [31]. The α- chain crosslinks also increase stiffness and thickness, decrease inelastic deformation and appear to promote red blood cell retention during clot contraction [27,33,34,35]. FXIIIa also catalyses the crosslinking of antifibrinolytic proteins such as fibronectin, plasmin inhibitor, thrombin activatable fibrinolysis inhibitor (TAFI), plasminogen activator inhibitor 2 and even the inflammatory protein complement C3 to fibrin, which further increases the clot’s resistance to fibrinolysis [36,37,38]. FXIIIa mediated crosslinking creates a fibrin scaffold that is capable of accommodating red blood cells, platelets and endothelial cells [33,39,40]. 

The fibrin network also interacts with platelets via a surface integrin receptor αIIbβ3 [41], and this enhances platelet aggregation [41]. As thrombin induces the fibrinogen-platelet interaction it simultaneously converts fibrinogen to fibrin, therefore, with time, polymerised fibrin becomes the ligand for activated platelet surface receptor αIIbβ3 [41]. This results in a platelet–fibrin meshwork that enables clot contraction, which occurs through the action of myosin II and actin proteins in the platelets [42]. The fibrin in the platelet–fibrin meshwork facilitates the transmission of force during contraction [41]. Clot contraction assists in the restoration of haemostasis by forming a temporary sealant to stem bleeding while restoring blood flow to the affected area by decreasing the clot’s size [42].

### 2.2. Clot Dissolution/Lysis

**Figure 3:**
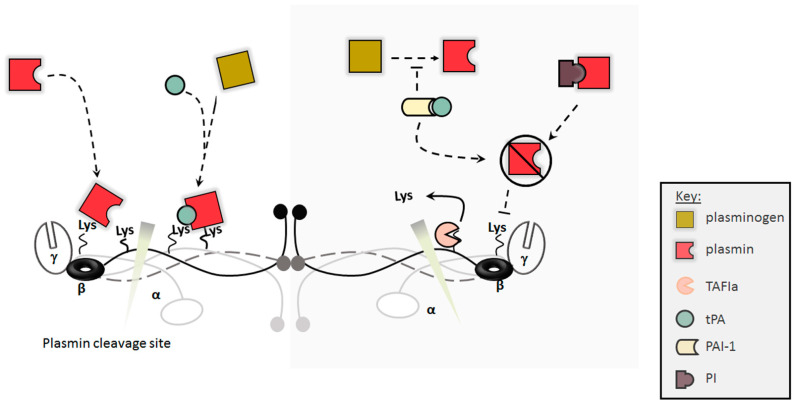
Fibrinolysis. Fibrin clots are broken down by plasmin, which is produced from plasminogen by the tissue plasminogen activator (tPA). Conversion of fibrinogen to fibrin results in the exposure of tPA and plasminogen binding sites. These sites propagate fibrinolysis by enabling the binding of plasminogen and tPA to fibrin network in close proximity, thus enhancing conversion of plasminogen to plasmin. Fibrinolysis is regulated by antifibrinolytic proteins including thrombin activatable fibrinolysis inhibitor (TAFI), plasmin inhibitor (PI) and plasminogen activator inhibitor (PAI-1). Activated TAFI (TAFIa) inhibits the lys-dependent interaction of plasminogen and tPA with fibrin, thus blocking fibrinolysis. PI interacts with Plasmin preventing plasmin-mediated cleavage of fibrin. PAI-1 interacts with tPA inhibiting the conversion of plasminogen to plasmin.

Fibrinolysis is a tightly regulated process that involves the dissolution of stable fibrin clot to reinstate normal blood flow (Figure 3) [43]. Although dissolution of clots is important in maintaining haemostasis, the spatial and temporal regulation of fibrinolysis activation is crucial to haemostasis. Fibrinolysis is initiated by the interaction of plasminogen and the tissue plasminogen activator (tPA) [44] synthesised by the endothelial cell, which can therefore control local clot formation/lysis [45] (Figure 3). The conversion of fibrinogen to fibrin results in the exposure of cryptic tPA and plasminogen binding sites on the α-C domain (Aα chain, residues 392–610) [43]. The colocalization of tPA and plasminogen on fibrin leads to 500-fold increase in catalytic efficiency of plasminogen activation compared unbound tPA [46,47]. More tPA and plasminogen binding sites are found in the D region on the Aα chain (residues 148–160), however, these binding sites have a lower affinity compared to sites on the αC domains [48,49]. The Aα chain cryptic sites are not specific to tPA or plasminogen and interact with both proteins with similar affinity, however, under physiological conditions, these sites are saturated with plasminogen due to higher plasma concentrations of the protein [50]. A tPA-specific binding site is located in the D region on the γ-chain (residues 312–324), which is 45 Å away from the D region on the Aα chain (residues 148–160) [51,52,53]. The two sites are close in proximity such that binding of tPA and plasminogen to these sites brings the two close together, facilitating the activation of plasminogen [54] (Figure 3). The initial degradation of fibrin by plasmin results in the exposure of more plamin(ogen) binding sites in the C-terminus region, which in turn propagate fibrinolysis [55]. The partial degradation products of fibrin enhance fibrinolysis because the fragments are better at stimulating the tPA-mediated activation of plasminogen than intact fibrin, thus supporting the premise of fibrinolysis as a partly self-activating process [56].

A number of proteins inhibit the fibrinolytic process including: (i) TAFI [43,57], which cleaves off C-terminal lysine residues from partially degraded fibrin, thus preventing the lysine-dependent binding of plasmin(ogen); (ii) Plasmin inhibitor, which is cross-linked into the fibrin network by FXIII and inhibits plasmin by forming stoichiometric complexes with the protein [46]; and (iii) PAI-1, which inhibits plasmin generation, thus reducing clot breakdown (Figure 3).

## 3. The Impact of Fibrin(ogen) Modifications and Plasma Levels on Fibrin Clots

As fibrinogen is the main building block for fibrin clots, variations and alterations in the molecule and its circulating levels in plasma have direct implications on clot formation, dissolution kinetics and the overall structure of the fibrin matrix [58]. The heterogeneity associated with fibrinogen molecules is influenced by environmental factors, genetic polymorphism, alternative mRNA splicing, proteolytic cleavage and post-translational modifications [59,60,61,62,63,64,65] (Table 1). Alterations in fibrin clot structure may result in either hyperfibrinolysis or hypofibrinolysis, both implicated in a number of pathologies. Deregulation of both clot formation and dissolution has profound clinical consequences associated with bleeding or thrombosis.

### 3.1. Changes in Fibrinogen Concentrations

A relationship between elevated concentrations of fibrinogen and risk of cardiovascular disease (CVD) has been repeatedly highlighted [101,102,103]. High fibrinogen levels influence clot density and rigidity through increased fibre and branch points [5,104]. Elevated plasma levels of fibrinogen in patients with increased risk of myocardial infarction may be partly assigned to the formation of more compact and stiffer clots [104]. An association of elevated levels of fibrinogen with enhanced fibrin formation, increased clot mechanical stability and increased resistance to lysis suggested a relationship between fibrin network changes with increased risk of thrombosis [66]. Conversely, low levels of fibrinogen are associated with increased bleeding due to less stable fibrin networks [3,21].

### 3.2. Post-Translational Modifications

Fibrinogen can undergo oxidation, nitration, glycosylation, phosphorylation, acetylation and homocysteinylation. It has been shown that fibrinogen is more susceptible to oxidation compared to other plasma proteins [58]. Post translational modifications of fibrinogen have a direct impact on clot formation, structure, and lysis [58]. Most of these modifications have been shown to result in fibrin clots that are less susceptible to lysis and have a high occurrence in disease states (Table 1). However, acetylation, following aspirin administration (a classical antiplatelet agent), is associated with clots that are easier to lyse, thus making aspirin an agent with a dual anti-thrombotic mode of action [58,91].

### 3.3. Genetic Polymorphism and Splicing

Genetic polymorphism and alternative splicing have given rise to different isoforms of fibrinogen. As mentioned earlier, alternative splicing results in the γ’ chain, which is 20 amino acids longer than the γ chain [105,106]. This C-terminal extension has a negatively charged region with thrombin and FXIIIa binding sites [107,108]. FpB cleavage from the fibrinogen heterodimer γ/γ’ has been shown to be slower and is implicated in delayed lateral aggregation, the formation of thinner fibres with more branch points and reduced clot pore size [70]. It has been shown that clots formed from γ/γ’ exhibit increased mechanical stiffness and resistance to fibrinolysis [71,109]. Although some studies demonstrated prothrombotic effects for γ/γ’, Omarova et al. has shown that γ/γ’ may be anti-thrombotic in venous thrombosis [110], indicating that the exact clinical significance of γ/γ’ remains an area for future research.

Alternative splicing also gives rise to a version of the α-chain, AαE, that is 236 amino acids longer resulting in fibrinogen molecule that is 420 kDa (Fib420). Fib420 α- chains have an additional globular domain that contains Ca^2+^-binding sites. Calcium ions promote the FXIII crosslinking of fibrin fibres and modulate the susceptibility to lysis by plasmin [72].

The single nucleotide polymorphism that substitutes arginine with lysine at position 448 in the Bβ chain C-terminus gives rise to clots with thinner fibres, smaller pores, increased stiffness and increased resistance to lysis [73]. This isoform is associated with thrombotic tendencies and coronary artery disease [111,112].

## 4. Implications of Changes in Fibrin Clot Characteristics in Disease States

The modulation of fibrin(ogen) such as post-translational modifications can either lead to hyperfibrinolysis or hypofibrinolysis, thus predisposing to bleeding or thrombosis.

### 4.1. Fibrin(ogen) in Bleeding Disorders

Fibrinogen levels and quality in plasma have been implicated in acquired and inherited bleeding disorders and are divided into two types, I and II [113]. Type I inherited disorders, including afibrinogenemia and hypofibrinogenemia, are associated with low concentrations of fibrinogen and treated efficiently with plasma-derived fibrinogen concentrate infusions [113,114]. However, despite low levels of fibrinogen, genetic mutations in a hypofibrinogenemia can result in a thrombotic phenotype [115]. Type II inherited disorders, such as dysfibrinogenemia and hypodysfibrinogemia, are a result of missense mutations in the Aα, Bβ and γ chains [113], causing dysfunctional fibrinogen [116]. In dysfibrinogenemia, most missense mutations affect fibrin polymerisation, resulting in variable tendency for bleeding. Hypodysfibrinogenemia manifests as a result of low fibrinogen concentrations in a dysfibrinogenemia state [117]. Dysfibrinogenemia and hypodysfibrinogenemia can have a vast array of clinical presentations ranging from asymptomatic, to bleeding and even to thrombotic tendencies [20,36]. A case study by Casini et al. revealed that the fibrin clot structures of individuals with a bleeding phenotype show increased clot permeability and thick fibrin fibres [117]. In contrast, individuals with thrombotic disorders had clots that were dense and displayed prolonged lysis [117].

Congenital deficiencies in coagulation factors VIII, IX and XI lead to bleeding disorders, such as haemophilia A and B. These deficiencies result in the inability to amplify and propagate thrombin production from prothrombinase [118]. The low levels of thrombin result in the slow activation of several other pro-coagulation proteins such as fibrinogen, FXIII and TAFI, which lead to the formation of stable clots [118,119,120]. Fibrin clots formed in haemophilic plasma are more soluble than normal because they have thicker fibres and larger pores, thus explaining the increased permeability [121,122,123]. The severity in bleeding in haemophilia is related to the degree of clot permeability, further emphasising the role of fibrin structure in predisposition to disease. Deficiencies in factor XIII result in a rare bleeding disorder [124] secondary to lack of fibrin fibre crosslinking and reduced incorporation of anti-fibrinolytic proteins such as PI [37,38,125,126,127], thus reducing clot stability [128]. Hyperfibrinolysis can be acquired through post-translational modifications of fibrinogen such as citrullination, which has been reported to inhibit thrombin-mediated fibrin polymerisation [89,90].

### 4.2. Fibrin(ogen) in Thrombosis

Thrombosis is a manifestation of hypofibrinolysis and is frequently seen in cardiovascular disease, partially related to more compact fibrin networks and partially to secondary changes in the fibrinolytic system [100,129,130,131,132,133]. Clots displaying such characteristics have been observed in pathologies such as cancer, diabetes and antiphospholipid syndrome, disease states that are associated with increased risk of thrombosis [134,135,136].

## 5. Pharmacological Therapies Targeting Fibrinogen and the Fibrin Network

Pro- and anti-coagulants and thrombolytic therapies have been used to modulate fibrin polymerization and dissolution. Some therapeutic strategies used for prevention and treatment of cardiovascular disease, including anti-platelet, anti-hyperlipidaemic, anti-hypertensive and glucose lowering agents are associated with changes in clot structure and lysis [137]. Mechanisms for changes in clot structure include post-translational modification of the protein (acetylation of fibrinogen by aspirin), alteration in FXIII activity (metformin) or modulation of plasma levels of fibrinogen and PAI-1 (statins) [138,139,140].

### 5.1. Thrombolytic Therapeutics

**Figure 4:**
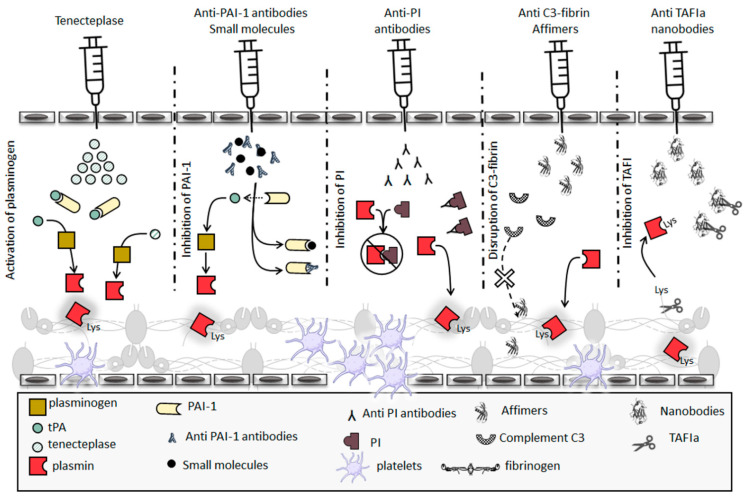
Current and potential thrombolytic therapies. The aim of thrombolytic drugs is to breakdown obstructive clots. Tenecteplase is a third-generation thrombolytic drug that has high specificity for fibrin and is available for clinical use. Due to complexities associated with use of tenecteplase, TAFI, PAI-1, PI and C3 are being explored as therapeutic targets. Small molecule and antibodies have been developed to inhibit PAI-1, which should enable more efficient plasminogen to plasmin conversion. Anti-PI antibodies interact with PI preventing its ability to block plasmin activity, thus enhancing clot lysis. C3 has been shown to interact with fibrin enhancing clot resistance to lysis, an effect that can be modulated with the use of Affimer technology. A panel of nanobodies has been found that inhibit TAFIa activation and activity, consequently facilitating lysis. While PAI-1, PI, C3 and TAFI- targeted therapies are exciting and offer a specific approach, none of these inhibitory agents made into clinical practice and future research in this area is required.

The purpose of thrombolytic drugs is to aid the degradation of obstructive thrombi by activating plasminogen (Figure 4). First generation thrombolytic drugs such urokinase and streptokinase were not fibrin specific, increasing bleeding complications [141].

In contrast, second generation thrombolytic drugs such as tPA and alteplase were fibrin-specific, however, high concentrations removed this specificity and still resulted in bleeding complications. Third generation thrombolytic drugs such as tenecteplase were developed to improve the half-life, specificity and to reduce the side effects [142]. Tenecteplase, a mutant variant of tPA, was shown to have 14-fold higher specificity for fibrin compared to alteplase, a longer half-life and slower clearance with an 80-fold increased resistance to inhibition by PAI-1 [143] (Figure 4). Although Tenecteplase has improved fibrin specificity, it is not devoid of side effects. In order to improve safety, novel delivery methods for ensuring fibrin specificity in plasminogen activators have been the focus of much research. Fibrin-targeting antibodies have been shown to enable the local enrichment at thrombus sites and increase potency of thrombolytic agents. These studies have shown success using in vitro and in vivo assays; however, none have reached clinical stages, probably due to the complexities associated with protein crosslinking and long-term stability [144].

Novel strategies have also involved the development of carrier-based systems and triggered release approaches such as fibrinolytic agent carrying erythrocytes, echogenic liposomes and fibrinolytic agent bearing nanoparticles [145,146,147,148]. However, further optimisation of these methodologies is required before these agents make it to the clinical arena.

Due to the general high risk of bleeding with thrombolytic agents, some research focused on inhibiting a specific antifibrinolytic protein such as TAFI, PAI-1 or PI. Several small molecule inhibitors against TAFI have been developed, however, as with most drug discovery and development strategies, a limited number progressed to clinical trials (phase I and phase II); unfortunately, these trials were discontinued due to the lack of selectivity and unwanted off-target reactivity [149]. Similarly, most small molecules developed against PAI-1 showed promise, but none reached a stage of clinical testing [150].

Other attempts involved inhibitory monoclonal antibodies and nanobodies against TAFI and PAI-1 [149]. Monoclonal antibodies and nanobodies have been reported to inhibit TAFI directly [151,152]. A panel of nanobodies that inhibit TAFI activation and activity via different modes were developed and shown to be effective using in vitro/in vivo studies [153,154] (Figure 4). Owing to the pleiotropic biological function of PAI-1, inhibitory antibodies tended to exhibit side effects. Since highly specific monoclonal antibodies against TAFI and PAI-1 had been raised, a bifunctional and bispecific antibody was developed [155]. Administering the heterodimer antibody into murine models resulted in significantly enhanced fibrinolysis without increased bleeding [155]. Even though a diverse pool of PAI-1 inhibitors has been developed and extensively characterised, only a few have recently proceeded to clinical trials [156] and results are awaited with interest.

The potential of PI as a therapeutic target is demonstrated by its inhibitory action once incorporated into a clot where it increases clot resistance to lysis [157]. Early PI inhibitory studies in rat models, using a pool of polyclonal anti-PI F(ab)2 fragments, demonstrated acceleration of fibrinolysis [158]. A more targeted approach that involved a monoclonal antibody raised against PI demonstrated significantly increased clot lysis [159] (Figure 4). The N-terminal domain of PI plays a key role in crosslinking to fibrinogen and therefore peptides mimicking this domain were developed and demonstrated competition with full-length PI in relation to incorporation into clots [160,161,162], and thus may help in facilitating lysis.

Complement C3 protein is crosslinked into the fibrin network and can also form noncovalent interactions with the clot [126,163], increasing clot resistance to fibrinolysis [125,163,164]. Moreover, an association of elevated plasma levels of C3 with increased clot resistance to lysis was observed in type II diabetes mellitus patients [156,157]. Although C3 is not yet considered an antithrombotic therapeutic target, disrupting the C3-fibrin interaction using Affimer technology (previously known as Adhiron) reduced clot lysis time [165] (Figure 4). This strategy demonstrated the potential value of using antibody mimetics in identifying interaction hotspots with therapeutic potential on fibrinogen. Our work has shown that fibrinogen-binding Affimers can block the interactions of fibrin(ogen) with other proteins that determine clot resistance to lysis, thus providing a unique opportunity to modulate thrombosis risk.

### 5.2. Hypofibrinolysis Therapeutics

**Figure 5:**
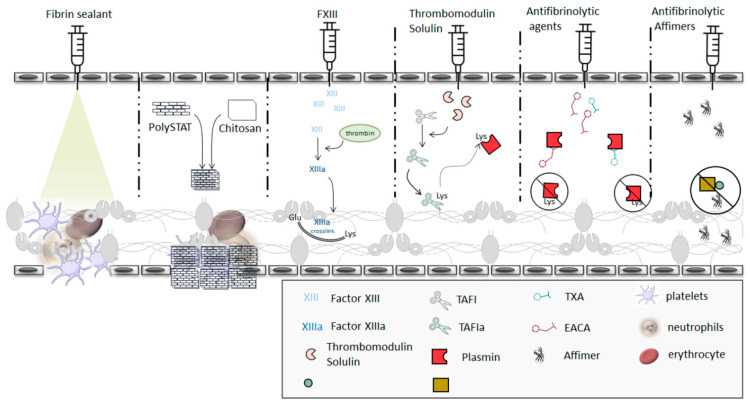
Current hyperfibrinolysis therapeutics and potential alternatives. Fibrin sealants are surgical haemostatic agents composed of a mix of pro-coagulation proteins, which ensure clot formation and prevents premature clot lysis. PolySTAT, a fibrin specific polymer with multiple fibrin domains, facilitates fibrin crosslinking while Chitosan, a biodegradable N-acetylglucosamine polymer, interacts with erythrocytes leading to erythrocyte agglutination. PolySTAT–Chitosan gauzes increase clot stability decreasing susceptibility to lysis. FXIII treatment results in fibrin crosslinking and crosslinking of PI to the fibrin network which improve clot stability and resistance to lysis. Solulin, a soluble form of thrombomodulin, acts by activating TAFI, which in turn cleaves lysine residues on fibrin preventing degradation of fibrin. Synthetic lysine analogues, TXA and EACA interact with plasmin(ogen) and block fibrin–plasmin(ogen) interactions, leading to increased resistance to clot lysis. More recently, Affimers, antibody mimetics raised against fibrinogen, were shown to interact with the fibrin, blocking plasmin-mediated degradation of the fibrin network. In vitro studies showed the potential use of Affimers in stabilizing the fibrin network and preventing premature lysis of the clot.

Bleeding complications that arise from traumatic vessel injury or bleeding disorders are characterised by unstable clots and are a major cause of morbidity and mortality. Figure 5 summarises the current and potential therapeutics for hyperfibrinolysis. In congenital bleeding disorder such as haemophilia, treatment strategies initially involved replacement of deficient factors. Although factor replacement is the chosen form of treatment, there are several issues such as the development immunogenicity that counteracts the purpose of replacements [166,167]. These issues have led to the development of strategies that bypass replacement and target the fibrin network. FXIII, thrombomodulin and tranexamic have been explored as fibrin network stabilising agents. Co-treatment with FVIII and FXIII enhances fibrin crosslinking and the incorporation of PI into the fibrin network [168]. Higher than normal concentrations of FXIII resulted in better stabilisation of clots made from haemophilia patients [168].

A number of studies on haemophilia have largely focused on the thrombin dependent pro-coagulation activity with minimal attention to the thrombin-dependent antifibrinolytic activation. Bleeding in haemophilia is in part due to enhanced fibrinolysis because of defective TAFI activation. Direct addition of TAFI to haemophilic plasma reduced clot lysis and stabilised the clot [169]. Moreover, the addition of thrombomodulin and/or TAFI to plasma of haemophilia patients who had developed inhibitory antibodies against FVIII decreased clot lysis [169].

Supplementing FVIII-deficient plasma with the soluble thrombomodulin solulin resulted in a four-fold increase in clot stability [170], a clear example of a new paradigm in which fibrin formation and maintenance are targeted in the development of therapeutics to reduce bleeding [170] (Figure 5).

Some studies have demonstrated that low levels of thrombin produced in haemophilia patients results in the formation of unstable clots that undergo premature lysis. Tranexamic acid (TXA) and epsilon amino caproic acid (EACA) are synthetic lysine analogues that act as antifibrinolytic agents by binding to plasmin(ogen) [171]. The TXA– or EACA–plamin(ogen) interactions block fibrin–plasmin(ogen), therefore preventing clot dissolution (Figure 5). TXA is used more widely because it is more potent and has a 6–10-fold higher affinity for lysine sites compared to EACA [172]. In a clinical trial study, the use of TXA as a monotherapy had no significant benefits [173], however, when used as an adjunctive, significant benefits were observed [174]. The combination of FVIII replacement therapy and TXA improves blood clotting parameters such as clot firmness when compared to FVIII replacement alone [175]. TXA can cross the blood–brain barrier and increase the risk of seizures, making dosage consideration in treatment critical [176]. It has also been found to prevent plasmin inhibition by PI and exhibit pro-fibrinolytic properties at high concentrations, further highlighting the complexities in developing fibrin-related therapeutic agents [55,177,178].

In addition to antifibrinolytics, fibrin sealants have been used to limit blood loss associated with trauma and surgery. Fibrin sealants are surgical haemostatic agents composed of a mix of proteins including fibrinogen, thrombin, FXIII and antifibrinolytic agents [179,180,181,182] (Figure 5). Fibrin sealants are used in a variety of surgical procedures and have multiple modes of action: clot formation, wound healing and gluing tissues together [6,179,180,181,183,184,185]. Although effective, there are a number of limitations associated with adopting fibrin sealants. The complexity associated with fibrin sealant preparation is potentially problematic for emergency situations [186]. When inadvertently injected intravascularly, the sealants increase the risk of thrombosis. Human-derived protein used in the sealants have been reported to cause anaphylaxis or infection [6]. Moreover, the recombinant proteins used in the sealants are not easily accessible, making them expensive [186,187], thus preventing widespread use.

An alternative for fibrin sealants, is an engineered haemostatic polymer (PolySTAT) [188] (Figure 5). PolySTAT is a polymer consisting of multiple fibrin-specific binding domains. It acts in a similar fashion to FXIII in that it crosslinks adjacent fibrin monomers through noncovalent bonds. Thromboelastography studies demonstrated that supplementing whole blood with PolySTAT accelerated clotting, increased clot strength and resistance to lysis [189]. Initial animal studies showed that intravenous administration of PolySTAT increased survival rate and decreased blood loss [189].

Our group has recently demonstrated the potential use of antibody mimetics that alter the fibrin network to control bleeding disorders [190]. Two high affinity fibrinogen-binding Affimers were found to prolong lysis of clots made from purified fibrinogen, plasma or whole blood (Figure 5). Interestingly, one Affimer induced severe changes to clot structure whereas the other maintained the physiological structure of the fibrin network [190]. Our data suggested that the antifibrinolytic effects of the Affimer that maintained physiological clot structure were related to disruption of tPA-plasminogen interaction on fibrin network [190]. When added to FVIII deficient plasma, the Affimer displayed a concentration-dependent delay in clot lysis time without affecting clot firmness [190]. This opens a new avenue in treating bleeding disorders by stabilising the fibrin network using Affimer proteins. Taken together, fibrinogen-binding Affimers represent a new tool to modify both thrombosis and bleeding potential through either prevention of anti-fibrinoytic protein interaction with fibrinogen, making the clot easier to breakdown, or through modulation of the tPA–plasminogen interactions on the fibrin network, thus increasing resistance to lysis. This may result in safer treatment strategies that maintain physiological haemostasis while addressing the pathological changes in coagulation proteins.

## 6. Conclusions

While our knowledge of the fibrinogen molecule has increased exponentially over the past few decades, the use of this knowledge for clinical therapeutic purposes has been generally limited. Powerful fibrinolytic agents have had an impact on managing patients with arterial or venous occlusions, but their role is limited due to the narrow therapeutic window, high risk of bleeding complications and the superiority of percutaneous coronary intervention for the treatment of myocardial infarction. Rather than using a “sledge hammer” approach for thrombotic vascular occlusion, which increases the risk of bleeding complications, it is perhaps safer to focus on specific molecules that interact with fibrinogen in order to facilitate clot breakdown while maintaining physiological haemostasis. The same approach can be adopted for bleeding disorders through employing agents that stabilise the fibrin network, thus avoiding the use of multiple coagulation factors that can potentially result in thrombotic complications.

Despite encouraging advances, a key drawback is the general lack of agents that directly target the fibrinogen molecule. The emergence of Affimers as small proteins that bind fibrinogen and control clot stability/resistance to lysis creates a new avenue that may prove to be clinically viable for the treatment of both bleeding and thrombotic disorders. The simplicity of using the same technology to develop agents for both bleeding and thrombotic disorders is particularly attractive. Naturally, these are early days, and there is a long way to go before fibrinogen-specific agents can be routinely used in clinical practice and future research in this area is awaited with interest.

## Figures and Tables

**Table 1:** The effects of fibrinogen modifications on clot structures.

Modifications	Associated Effects
Elevated Fibrinogen concentration	↑ clottability, ↑ clot density, ↑ resistance to clot lysis [5,66]
High Thrombin concentration	↑ fibre diameter, ↑ clot density, ↑ resistance to clot lysis [67,68]
Fibrinogen polymorphisms and splice variants	γ’:↑ clot stiffness, ↓ fibre thickness, ↓ clot permeability, ↑ clot density, ↑ resistance to clot lysis [69,70,71] Fibrinogen 420: ↓ fibrin degradation by plasmin, ↑ resistance to clot lysis [72] BβArg448Lys: ↓ fibre diameter, ↓ permeability, ↑ clot stiffness, ↑ resistance to fibrinolysis [73]
Oxidation	↑ clottability, ↓ fibre thickness, ↓ clot stiffness, ↓ clot permeability, ↑ clot density, ↑ resistance to clot lysis [74,75,76,77]
Glycation	↑ clottability, ↓ clot permeability, ↑ clot density, ↑ resistance to clot lysis [78,79,80,81,82,83]
Phosphorylation	↓ fibre thickness, ↓ resistance to clot lysis [84,85,86,87]
Citrullination	↓ clottability, ↓ fibre thickness, ↓ clot density, ↑ lysis [88,89,90]
Acetylation	↑ fibre thickness, ↓ clot stiffness, ↑ clot permeability, ↓ clot density, ↓ resistance to clot lysis [83,91,92]
Homocysteinylation	↑ clot density, ↑ resistance to clot lysis [93,94]
Guanidinylation	↓ fibre thickness, ↓ clot permeability [95]
Carbamylation	↓ fibre thickness, ↑ clot density, ↓ crosslinking, ↑ resistance to clot lysis [96]
Nitration	↑ clot stiffness, ↑ resistance to clot lysis [97,98]
Aspirin	See acetylation
Metformin	↓ crosslinking, ↓ resistance to clot lysis [99]
Elevated Lipoprotein concentrations	↓ clot permeability, ↑ resistance to clot lysis [100]
